# Impact Strength of Adhesive Joints of Pre-Impregnated Composite Elements

**DOI:** 10.3390/ma18122887

**Published:** 2025-06-18

**Authors:** Andrzej Komorek, Paweł Żeglarski, Jan Godzimirski

**Affiliations:** 1 Department of Aviation, Polish Air Force University, 08-530 Deblin, Poland; 2 43rd Air Naval Base, 81-197 Gdynia, Poland; pawel.zeglarski@gmail.com; 3 Department of Mechatronics and Aviation, Military University of Technology, 00-908 Warszawa, Poland; jan.godzimirski@wat.edu.pl

**Keywords:** polymer composite, lap joints, impact strength

## Abstract

Composite materials’ contribution to the construction of manned and unmanned aircraft continues to grow and, together with the increased use of these materials, there is a growing need to develop an optimal method of joining composite components and carrying out repairs following operational damage. One such method is bonding by means of adhesive bonds, many of whose properties are already quite well known. However, relatively little is known about the impact strength of adhesive joints in general, including adhesive joints of composite components. This paper presents a concept for conducting such tests using adhesive lap joints. The sample pieces were cut from a 9-layer, 2 mm thick composite panel made with an autoclave technique. The results show that the lowest impact strength and shear strength occurred for adhesive joints made with an epoxy adhesive with the highest Young’s modulus. The best results were obtained for the adhesive whose joints became destroyed in equal proportions in a cohesive–adhesive manner.

## 1. Introduction

Ever since the appearance of first aircraft structures, a wide range of construction materials has been used, such as wood, steel, leather, aluminum alloys, titanium, and composites. Over the years, the proportion of the above-mentioned materials has varied. Structural composites emerged in the 1950s and 1960s of the 20th century. Over the past decade or so, the aerospace industry has seen a significant increase in the use of composites, to the point where they have become the primary structural material of today’s aircraft [1,2,3,4]. Among composite materials, carbon-fibre-reinforced polymer matrix composites are currently used most commonly [5,6,7], mainly due to the high strength and stiffness of components made from this material, which are higher than those of glass-fibre or aramid-fibre reinforcements [8,9,10].

In the context of the distinctive characteristics of composites, an important problem lies in their combination, both with each other and with other materials [11,12]. Well-known technologies applicable to joining metals, such as sealing and welding, cannot be used to join composites with curable polymer matrices. These constraints lead designers to opt for two types of connection. The first of these, which has been in use for decades, is mechanical connections, where riveting seems to be the most popular. The evolution of mechanical joints, as evidenced by the rivet nuts that are gaining in popularity, has made it possible to control pressure during the riveting process and minimize the stress on the carrier material. Although obtaining such features has allowed riveting to be used more widely, it is not able to ensure structural continuity in every case and counteract the phenomenon of stress concentration [13,14].

Adhesive joints have proved to be a solution to these problems [15,16,17,18,19,20]. The need to join composite materials is also related to the very process of using composite elements or structures and occurs when they are damaged. The only known method of repairing classic composites is glueing patches, which, although it does not recreate the original structure of the composite, restores its operational properties [21]. This is very important in the context of use, because structures made of layered composites, despite their high strength and low weight, are very sensitive to low-energy impact loads. If an unexpected load of this type occurs during the operation of an aircraft or other composite structure [22] (e.g., accidental dropping of a tool on an airframe element), the damaged element can be restored to operational efficiency only by glueing a patch [23]. Although making an adhesive bond often requires special surface preparation and the components to be bonded must fit well, limiting interference with the composite structure yields positive results. The large-scale use of adhesive joints in industry was made possible by the development of a methodology for their strength testing [24]. However, in relation to the well-developed methods for testing the static properties of adhesive joints, their impact resistance is still an object of research and discussion [25,26,27]. There are two normative impact test methods; however, they are dedicated to adhesive joints of metal components [28,29,30]. In addition, the above-mentioned methods are not very popular due to their limitations [31,32,33]. None of the above methods makes use of lap joints, which appear to be the most popular in static and fatigue testing. It is possible to come across examples of the use of such joints in impact testing of metal components. Due to the popularity of polymer composites and the adhesive joints used to connect them, the authors felt that it was appropriate to carry out a dynamic study of lap joints of composite components made with various structural adhesives, because such loads are inherent to normal operation of means of transport [34,35] as well as adhesively bonded ballistic shields [36,37].

## 2. Methodology of Research

Given the high proportion of composite materials and, in particular, carbon-reinforced composites used in modern aircraft structures, such a material was considered the most appropriate for the manufacture of test samples. An autoclaved composite plate [38], consisting of a 9-layer carbon prepreg, was commissioned from a professional company. The final result was composite panels measuring 1200 × 900 × 2 mm. The 2 mm thickness of the board was determined by the technical capabilities of the testing machine used for the adhesive impact tests.

GG204T IMP 503 (Impregnatex Compositi S.r.l., Castano Primo, Italy) carbon prepreg was used for the control panels. The matrix of the exploited semi-finished material is epoxy resin cured at temperatures ranging from 100 °C to 150 °C, while the reinforcement was 9 layers of 204 T carbon fabric with the properties shown in Table 1.

The basic strength properties obtained from the composite manufacturer are shown in Table 2.

The IMP 503 system used can be cured in a wide temperature range from 10 °C to 150 °C. Table 3 shows the glass transition temperature values for selected temperatures and curing times.

During the production of the composite, the initial pressure in the autoclave was set at 0.06–0.07 MPa. Then, heating was started with a gradient of 2–5 °C/min until reaching 120 °C. These conditions were maintained for an hour. After this time, cooling to 60 °C was started. After cooling to ambient temperature, the bag was opened, and the finished composite plate was removed from the autoclave.

**Figure 1:**
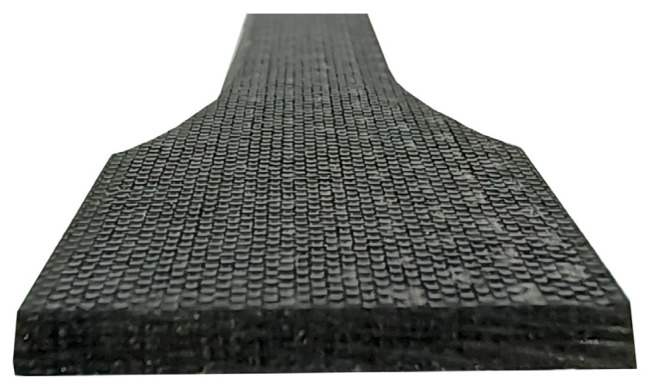
View of the composite surface.

The authors decided to join the samples without using any surface treatment on the parts to be joined, and the rough surface was achieved by using a delamination fabric—the composite contractor decided to use PS90 delamination fabric (Figure 1).

Prior to the testing of adhesive joints, the basic strength properties of the composite were determined experimentally. For this purpose, a static tensile test was carried out. For its execution, paddle-shaped B1 samples with a length of 150 mm were cut using water-jet cutting technology. The obtained samples were characterized by the uneven cut edges, which were removed using sandpaper with a gradation of 80.

In order to determine the static strength of the composite, the samples were tested in a tensile test using a Zwick&Roell Z100 machine (Zwick GmbH & Co., Ulm, Germany), based on the EN ISO 527-3:2018 standard [40]. Each batch was composed of five pieces. The test results are shown in Table 4.

The tensile strength value quoted by the composite manufacturer was similar to that obtained experimentally, while the Young’s modulus value determined experimentally was equal to 81.7 GPa against the 60 GPa specified by the composite manufacturer.

### Preparation of Samples for Joint Strength Testing

Rectangular plates measuring 70 × 20 mm were cut from the composite panels to make the overlapping glue joints. Again, as with the tensile test samples, the plates were characterized by non-uniformity of the cut edges, which prevented them from being fixed in the test machine grip handle. The inaccuracy was removed using 80-grit sandpaper (Mirka, Jeppo, Finland).

Before bonding, all the samples were thoroughly degreased and washed of carbon dust residues resulting from grinding. For this purpose, 99.9% isopropyl alcohol was used. After drying the samples in the laboratory drying chamber (temperature 50 °C, time equal to 120 s), they underwent bonding.

Six different adhesives differing in chemical composition, curing, and mechanical properties were used to bond the samples:

- Teroson PU9225 (Henkel AG & Co. KGaA, Dusseldorf, Germany) (two-component polyurethane-based adhesive);
- Henkel HY4080 (Henkel AG & Co. KGaA, Dusseldorf, Germany) (cyanoacrylate/acrylic hybrid structural adhesive);
- Henkel EA9497 (Henkel AG & Co. KGaA, Dusseldorf, Germany) (two-component epoxy adhesive);
- Henkel EA9464 (Henkel AG & Co. KGaA, Dusseldorf, Germany) (two-component epoxy adhesive);
- 3M DP420 (3M, Saint Paul, MN, USA) (epoxy structural adhesive recommended for bonding parts made with carbon fibre);
- Epidian 57 + hardener Z1 (Sarzyna Chemical Sp.z.o., Sarzyna, Poland) (epoxy composition mixed 10:1 by weight).

**Figure 2:**
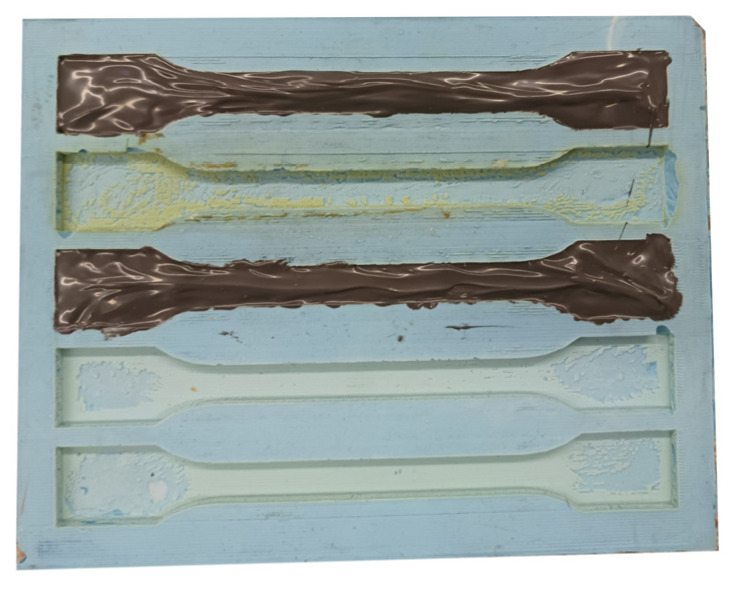
Samples cast in a silicone mould.

In order to determine the basic mechanical properties of the adhesives used in the study, paddle-shaped samples were cast from each adhesive (Figure 2).

The filled moulds were left until the adhesives cured under room conditions in accordance with the recommendations of the manufacturers of the individual adhesives.

**Figure 3:**
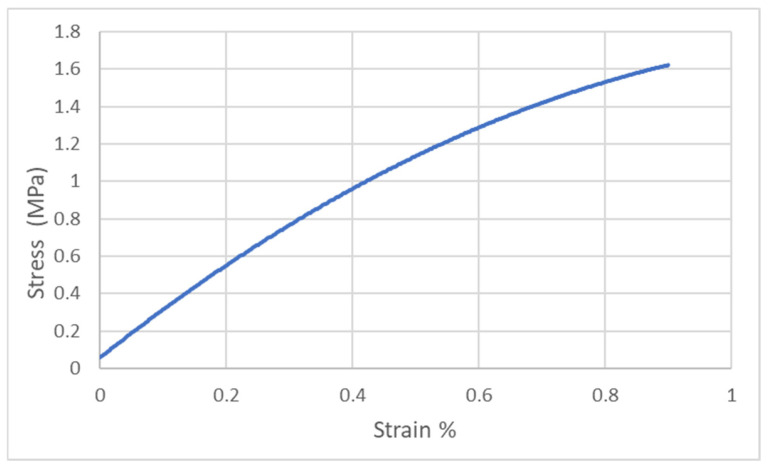
Static tensile test graph for PU9225 adhesive.

Each of the cast samples was cleaned of silicone residue and, where necessary, sanded to standardize the thickness and width of the samples. In order to determine the Young’s modulus of the adhesives, samples were stretched on the HT-2402 Hung Ta universal testing machine with a measurement range of up to 100 kN. An extensometer Epsilon 3542 with a measuring base of 25 mm was used for the tests. An example of a tensile test graph (PU9225 adhesive) is shown in Figure 3, (the tensile charts for the other adhesives are in the Supplementary Materials). The determined Young’s modulus values are shown in the table (Table 5).

## 3. Testing of Adhesive Joints

### 3.1. Sample Preparation

Fifteen samples were prepared for each adhesive (ten samples for impact testing and five for static tensile testing). Prior to bonding, pairs of composite plates were numbered, and the thickness of each plate was measured for subsequent determination of joint thickness.

PU9225 adhesive was used to glue the first batch of the samples together. In accordance with the manufacturer’s recommendations, apart from degreasing, the bonded surface required the application of the Teroson 150 primer (Henkel AG & Co. KGaA, Dusseldorf, Germany). Then, 15 min after varnishing with the primer, the glue was applied with a wooden spatula. The glue was being prepared as needed by manually mixing the ingredients. This action was determined by the short curing time of the glue, which prevented the use of a dedicated static mixer due to the rapid clogging of the nozzle. After the initial manual pressing of the samples, they were stacked one on top of one another in metal grips. To avoid accidental sticking, once the excess glue had flowed off, the samples were separated from each other using PET film.

**Figure 4:**
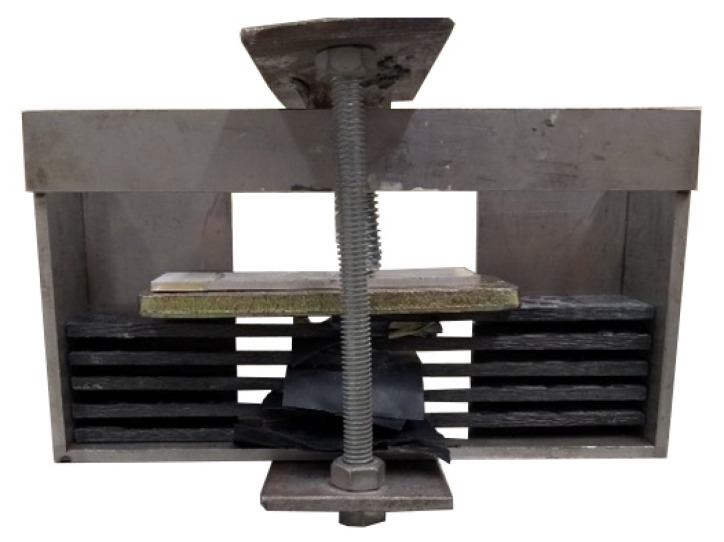
Samples fixed in the gripping handle.

Having placed five consecutive samples in the gripping handle, a yoke was applied to press the samples against the base (Figure 4). The M8 threaded clamping bolt in each batch of samples was tightened to a torque of 5 Nm, determined with a torque wrench (clamping force of approximately 1750 N), in order to achieve joints of the same thickness.

The HY4080GY hybrid adhesive, prepared by means of a dedicated static mixer, was used to produce the second batch of the samples.

The third batch used the EA9497 adhesive. As with PU9225, the adhesive cured very rapidly, necessitating manual mixing.

A fourth batch of samples was glued together using the EA9464 adhesive. Despite the fact that the curing time allowed a convenient use of the mixer, it was decided to prepare the adhesive composition manually. This was mainly due to the relatively high difference in density of the components, so that the final composition at the nozzle mouth did not have the desired homogeneity. In addition, each time the adhesive was applied to the plates, greater precision was required to achieve a thin layer than in the case of other adhesives due to the relatively high density of the adhesive.

The fifth batch used the 3M DP420 adhesive with a static mixer to speed up the process of applying the adhesive to the surface of the plates. Of all the adhesives used, this one had the easiest spreading, as well as the ease of achieving the desired thickness of the applied layer. For the last batch, the authors used the Polish adhesive Epidian 57 cured with the Z1 hardener. In this case, it was necessary to mix the resin and hardener by hand at a ratio of 1:10.

Once the bonding was complete, all the samples were left in the gripping handles for a minimum of 10 days at room temperature, which meant that the conditions specified in the manufacturers’ data sheets for each of the adhesives were satisfied. After this time, the samples were removed from the gripping handles, and a visual inspection of the quality of the joints was carried out and, if necessary, mechanical machining using a file or sandpaper was carried out in order to remove excess glue.

### 3.2. Tensile Strength Test

**Figure 5:**
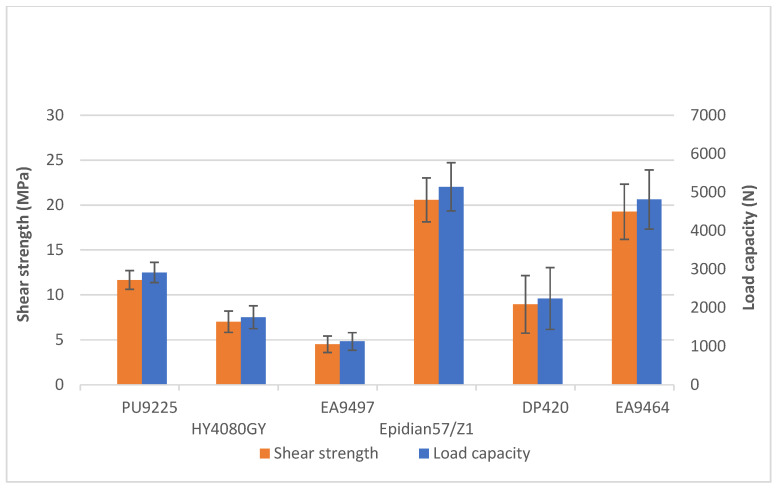
Shear strength and load capacity of test samples.

Impact testing of lap joints was preceded by determining their tensile shear strength. The tests were carried out using a Zwick&Roell Z100 universal testing machine (Zwick GmbH & Co., Ulm, Germany). The traverse movement was 2 mm/min time during the examination. Five tests were performed for each adhesive, the average strength values of which are shown in the graph (Figure 5).

The highest average shear strength (20.58 ± 2.45 MPa) was obtained for samples bonded with the Epidian 57/Z1 adhesive composition, while the lowest one was bonded with the EA9497 adhesive (21% of the strength of Epidian 57/Z1). The other tensile strength values were, respectively, 56% (PU9225), 34% (HY4080GY), 43% (DP420), and 94% (EA 9464) of the strength obtained for the Epidian 57/Z1 adhesive. The largest scatter of results occurred for the 3M DP420 adhesive, which was unexpected as, of all the adhesives used, this one had visually the most homogeneous texture and colour when mixed with a static mixer. In addition, its application process was the easiest and allowed the adhesive layer to be spread very evenly over each component to be glued.

### 3.3. Investigation of Impact Strength

**Figure 6:**
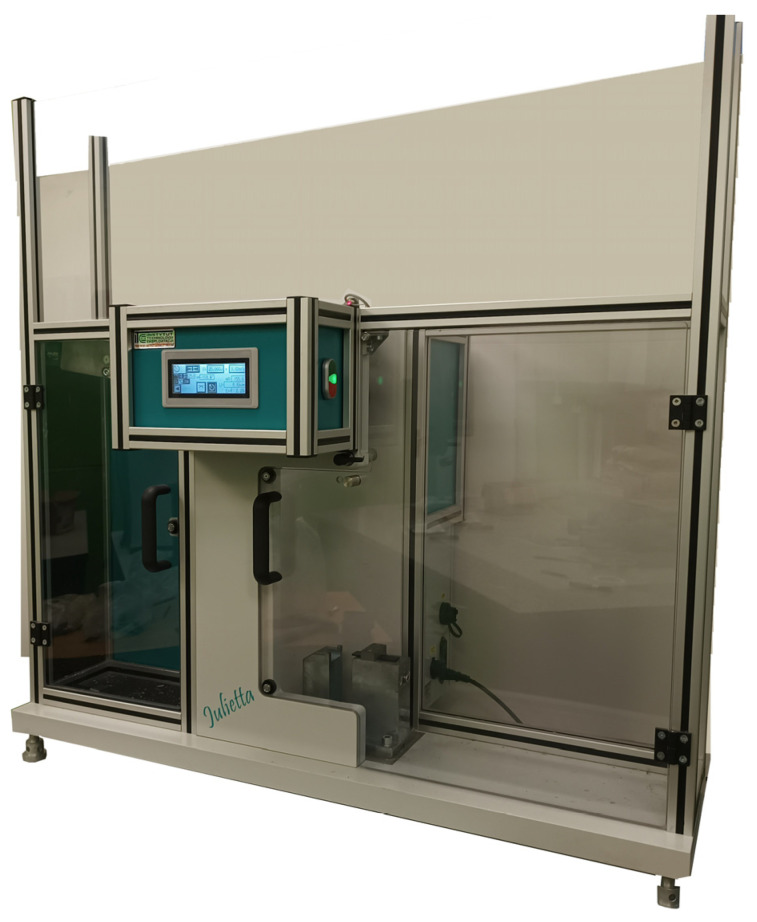
Julietta testing device.

Impact tests were carried out using the Julietta device (Łukasiewicz Research Network—Institute for Sustainable Technologies, Radom, Poland) (Figure 6) manufactured by Radom Institute of Technology and Exploitation with an installed pendulum of the maximum energy equal to 25 J and a velocity equal to 3.82 m/s.

Prior to the start of the tests, the yoke with the wedge and one sample plate (items rejected by the pendulum energy) were weighed in order to correct the results. The weight of the discarded items was 107.9 g.

**Figure 7:**
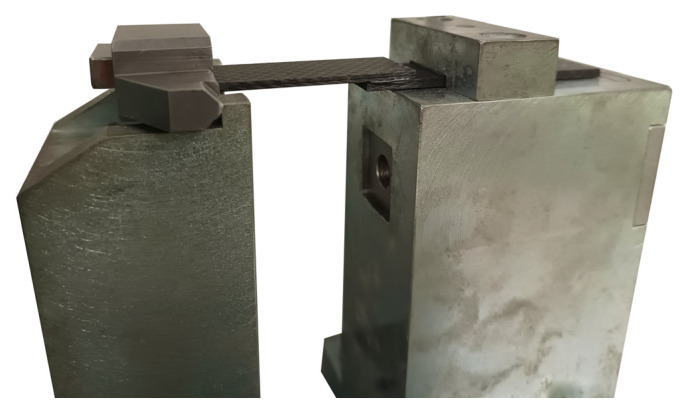
Sample fixed in a gripping handle, tightened with a screw.

One end of the sample was fixed inside the yoke with a wedge. The other end of the sample was fixed in a knurled holder and pressed down with a screw (Figure 7).

Despite the different consistency of the adhesive masses, the obtained joints were characterized by a similar thickness of approximately 0.02 mm.

**Figure 8:**
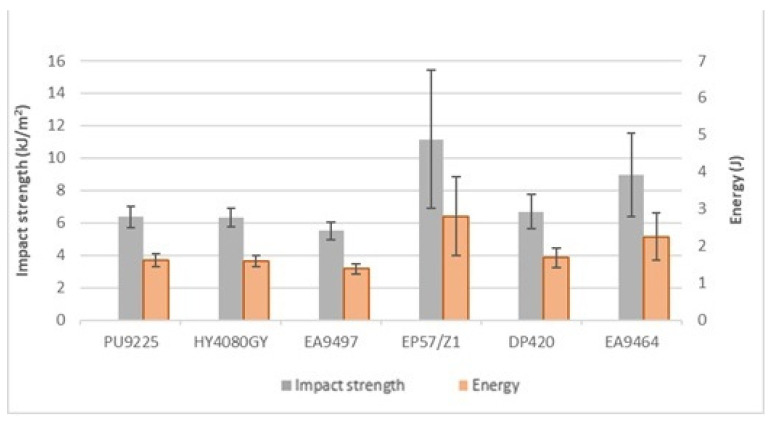
Comparison of mean values of impact strength and fracture energy of the tested adhesive joints.

Each batch included 10 samples, previously seasoned for at least 10 days. The obtained results of impact strength testing are shown in the graph (Figure 8).

The highest impact strength values were obtained for samples joined with the Epidian 57/Z1 composition. The average result for this adhesive was 11.48 ± 2.96 kJ/m^2^. The joints made with the EA9497 adhesive had the lowest impact strength values, with an average impact strength of 5.5 ± 0.56 kJ/m^2^, thus representing 49% of the impact strength of Epidian 57/Z1. The impact strength of the joints made with the other adhesives was as follows: 57% (PU9225), 56% (HY4080GY), 60% (DP420), and 80% (EA9464) of the impact strength joints made for the Epidian 57/Z1 adhesive.

The scatter of impact test results for joints made with the 3M DP420 adhesive was significantly lower than in static tests.

The obtained results for joints made with PU9225, HY4080, and DP420 adhesives proved to be similar, despite completely different chemical compositions that determine their assignment to different types of adhesives. The scatter of results for the HY4080GY and EA9497 compositions was the smallest of all the adhesives used, which, despite their low impact strength, allows the most reliable prediction of the behaviour of the joint under dynamic loads. The standard deviation of the results obtained for the joints made with the Epidian 57/Z1 adhesive was high and had a similar value to the one found in the studies of adhesive joints of block metal components [41].

In static and dynamic tests on samples of identical dimensions, made of the AW 2024T3 aluminum alloy (the surfaces of which were blasted and washed with petrol) glued with the Epidian 57/Z1 adhesive, for a similar joint thickness (approximately 0.02 mm), shear strength of 13.15 ± 1.14 MPa, and a dynamic destruction energy of 1.71 ± 0.13 J were obtained, i.e., much lower values than those obtained for joints of composite elements, where the average destruction energy was 2.87 ± 0.74 J.

The results of static and dynamic tests do not indicate any relationship between the static strength of the tested joints and their impact strength; a similar lack of relationship was also noted during tests of adhesive joints of aluminum alloy elements [26]. The obtained results also do not confirm the relationship between the Young’s modulus of the adhesives used and the impact strength of the composite element joints made with their use. In tests of adhesive joints of block metal elements, the joints made with the use of adhesives with higher Young’s modulus values showed lower impact strength values [42].

### 3.4. Visual Inspection of the Nature of the Damage

**Figure 9:**
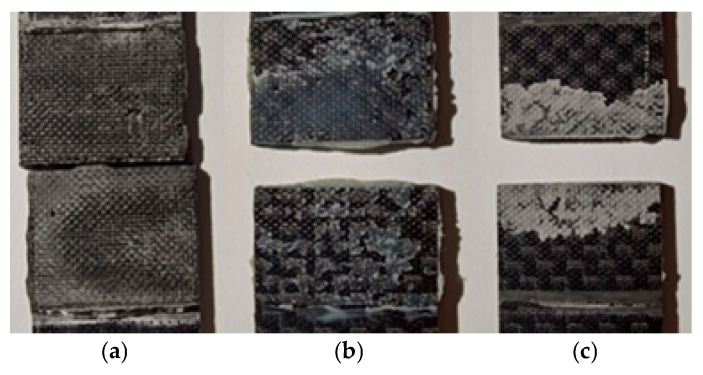
Breakages of adhesive-bonded samples after static shear strength testing: (**a**) adhesive PU9225, (**b**) adhesive HY4080GY, (**c**) adhesive EA9497.

**Figure 10:**
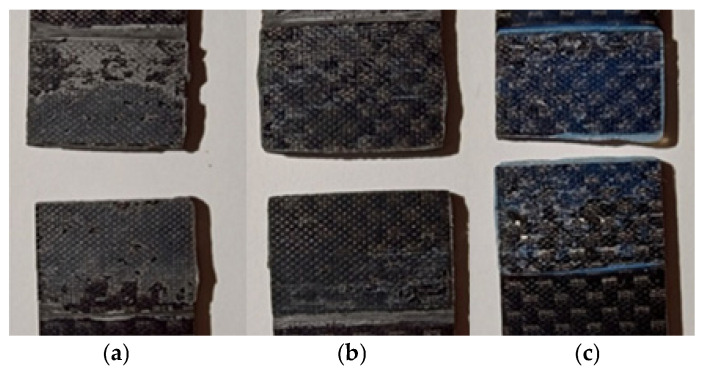
Breakages of adhesive-bonded samples after static shear strength testing: (**a**) adhesive PU9225, (**b**) adhesive 3M DP420, (**c**) adhesive Epidian 57/Z1.

**Figure 11:**
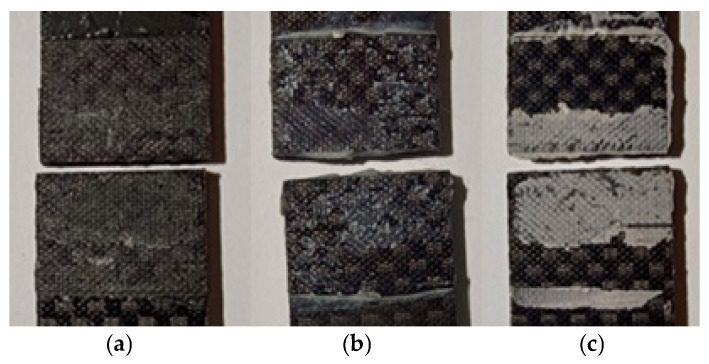
Nature of damage in the impact test of adhesive joined samples: (**a**) adhesive PU9225, (**b**) adhesive HY4080GY, (**c**) adhesive EA9497.

**Figure 12:**
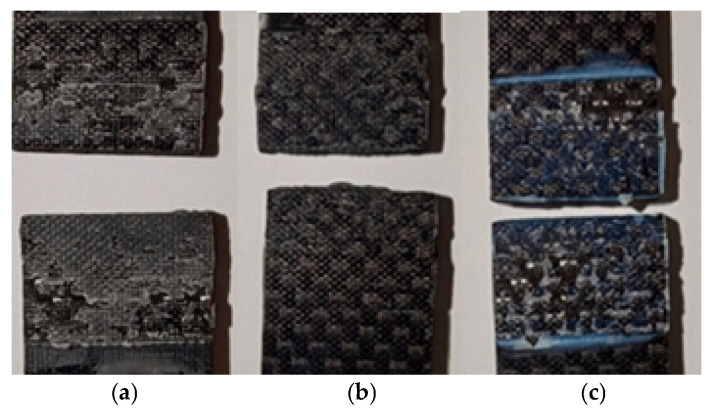
Nature of damage in the impact test of adhesive-bonded samples: (**a**) adhesive EA9464, (**b**) adhesive 3M DP420, (**c**) adhesive Epidian 57/Z1.

In order to determine the nature of the damage to the samples subjected to impact and tensile strength testing, selected impact fractures were visually inspected. The first set of images (Figure 9 and Figure 10 shows cracks resulting from the tensile test of the samples (static adhesive joint shear), while the second set (Figure 11 and Figure 12) shows samples subjected to impact tests. The breakages of samples bonded with the PU9225 adhesive had an adhesive nature of destruction (Figure 9a).

The samples bonded with the HY4080GY adhesive (Figure 9b) exhibited both cohesive and adhesive failure modes. In contrast, the samples bonded with the EA9497 adhesive (Figure 9c) showed poor adhesion between the glue and the material surface.

The nature of the damage of the samples bonded with the EA9464 adhesive (Figure 10a) was adhesive–cohesive.

Samples bonded with the 3M DP420 adhesive (Figure 10b) were characterized predominantly by adhesive failure across most of the fracture area. It was estimated that cohesive damage accounted for no more than 10%.

In the case of samples bonded with Epidian 57/Z1 (Figure 10c), the failure mode was a combination of adhesive and cohesive damage, with both types occurring in approximately equal proportions.

The nature of the impact failure in joints bonded with the PU9225 adhesive (Figure 11a) was predominantly adhesive, similar to the failure observed inshear strength tests. Nevertheless, the proportion of cohesive-type damage was slightly higher than in static tests.

Impact fractures of samples bonded with the HY4080GY adhesive (Figure 11b) were characterized by cohesive damage, in contrast to the adhesive failure observed in the static tests. Impact breakages of the samples joined with the EA9497 adhesive (Figure 11c) also showed poor adhesion of the glue to the bonded surfaces.

Impact fractures of samples bonded with the EA9464 adhesive (Figure 12a) were characterized by cohesive and adhesive damage. This was in contrast to the failure patterns observed in static tensile tests, where the majority of the sample surfaces were characterized by adhesive damage.

In the case of samples joined with the 3M DP420 adhesive, the impact breakages indicate cohesive damage, while in static tests the failures were adhesive in nature (Figure 12b).

The impact damage in samples bonded with with the Epidian 57/Z1 composition (Figure 12c) was characterized by an equal proportion of adhesive and cohesive failure, consistent with the failure modes observed in the static tests. This pattern was also similar to that obtained during impact testing of adhesive lap joints made from the AW 2024T3 aluminum alloy [26].

A summary of the damage characteristics observed is presented in Table 6.

The EA9497 adhesive showed the worst adhesion properties to the surface of the tested composite. The adhesive is also characterized by high stiffness, which, in total, resulted in the lowest static strength and impact strength of the joints made with it. A higher proportion of cohesive damage was found in dynamic tests than in static tests.

## 4. Conclusions

Based on the obtained results, the following conclusions were formulated:

(1) When selecting an adhesive for a joint that may be subjected to impact loads, it is essential to know the dynamic properties of such a joint. The impact strength of adhesive joints should not be predicted based on their static properties, because there is no clear relationship between these properties;
(2) Among the adhesives used in the study, composite samples joined with Epidian 57/Z1 had not only the highest impact strength but also the highest static strength. Furthermore, cracks in samples joined with Epidian 57/Z1 after both static and impact tests were characterized by adhesive and cohesive damage in equal proportions, which indicates the good quality of the connection regardless of the nature of the applied loads;
(3) Lap joints of composite components have a higher impact strength than those of aluminum alloy components;
(4) Joints made with EA9464 adhesive, despite having a slightly lower static and impact strength than Epidian 57/Z1 adhesive (but with a similar Young’s modulus value), have a significantly lower standard deviation, which allows a more reliable prediction of the behaviour of adhesive joints under loads;
(5) The lowest impact strength and shear strength occurred for adhesive joints made with epoxy glue with the highest value of Young’s modulus, similar to the impact testing of aluminum alloy component joints;
(6) Poor adhesion of adhesives to bonded surfaces can result from not subjecting the joined surface to additional blasting or abrasive treatment and also because of possible residues of the delamination fabric.

## Figures and Tables

**Table 1:** Properties of the reinforcement fabric.

Symbol	Unit Weight (g/m)	Weave	Fibre/Bundle		Thickness (mm)
			Matrix	Thread	
204T	204	Twill 2 × 2	Carbon 200 tex	Carbon 200 tex	0.20

**Table 2:** Basic properties of laminate cured at 130 °C for 60 min.

Mechanical Property	Test Standard	Value
Bending strength (MPa)	DIN EN 2562:1997-05 [39]	850
Modulus of elasticity in bending (GPa)	DIN EN 2562:1997-05 [39]	54
Tensile strength (MPa)	EN ISO 527-3:2018 [40]	550
Young’s modulus (GPa)	EN ISO 527-3:2018 [40]	60

**Table 3:** Example values of the glass transition temperature for different temperatures and curing times.

Curing Temperature	Curing Time	TG (°C)
100 °C	4 h	95–105
120 °C	1.5 h	110–115
130 °C	1.5 h	120–130

**Table 4:** Results of the static tensile test of the laminate.

Parameter	Sample					Average
	1	2	3	4	5	
Young’s modulus (MPa)	83,600	82,400	81,100	80,700	80,800	81,700 ± 1260
Tensile strength (MPa)	537	569	526	536	513	536 ± 21
Elongation (%)	0.988	1.100	0.976	0.927	0.849	0.969 ± 0.093

**Table 5:** Young’s modulus of the adhesives used in the study, determined experimentally.

Adhesive	Young’s Modulus (MPA)
PU9225	290
EA9497	5430
HY4080GY	2350
Epidian 57/Z1	1830
3M DP420	3210
EA9464	2400

**Table 6:** Nature of failure of examined connections.

Adhesive	Static Tests	Dynamic Tests
PU9225	adhesive	adhesive, 10% cohesive
HY4080GY	adhesive–cohesive	cohesive
EA9497	adhesive	adhesive
EA9464	adhesive–cohesive	cohesive/adhesive
3M DP420	adhesive (10% cohesive)	cohesive
Epidian 57/Z1	adhesive–cohesive	adhesive

## Data Availability

The original contributions presented in this study are included in the article/Supplementary Materials. Further inquiries can be directed to the corresponding author.

## Supplementary Materials

The following supporting information can be downloaded at: https://www.mdpi.com/article/10.3390/ma18122887/s1, Figure S1: Static tensile test graph for HY4080GY adhesive; Figure S2: Static tensile test graph for EA9497 adhesive; Figure S3: Static tensile test graph for Epidian 57/Z1 adhesive; Figure S4: Static tensile test graph for 3M DP420 adhesive; Figure S5: Static tensile test graph for EA9464 adhesive.
