# Supplementary material for: Impact Strength of Adhesive Joints of Pre-Impregnated Composite Elements
Source: Materials (Basel). 2025 Jun 18;18(12):2887. doi: 10.3390/ma18122887 (PMC12195519; doi:10.3390/ma18122887)
Supplement: Supplementary file 1 [file materials-18-02887-s001.zip › materials-3611542-supplementary.pdf]

The drawings of the adhesive tensile diagrams removed from the first version of the submitted manuscript are shown in Figures S1–S5.

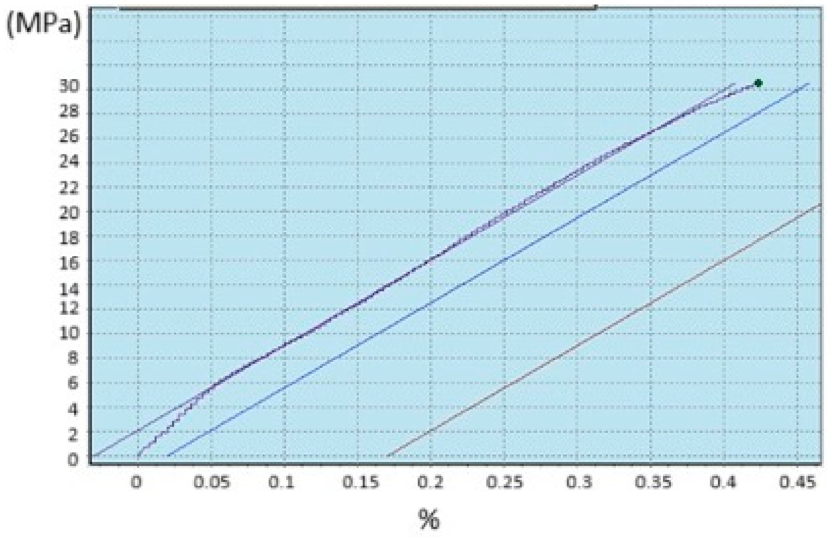


**Figure S1.** Static tensile test graph for HY4080GY adhesive.

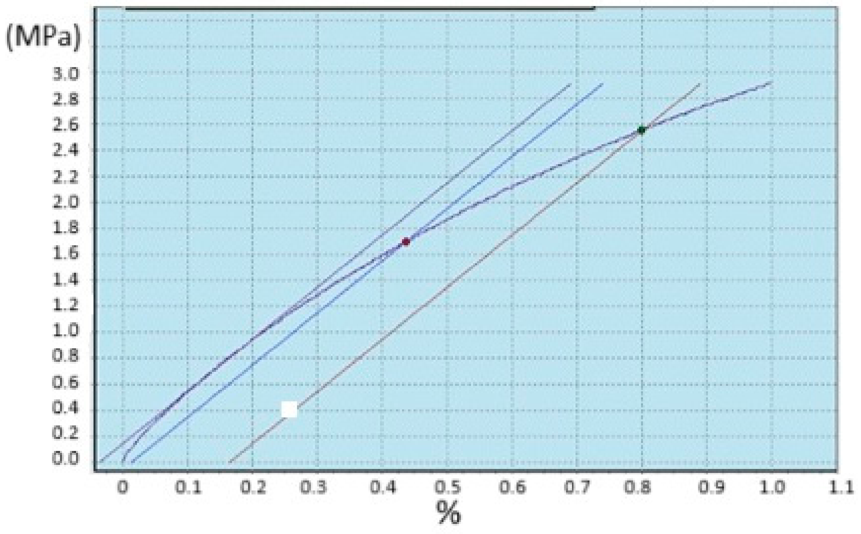


**Figure S2.** Static tensile test graph for EA9497 adhesive.

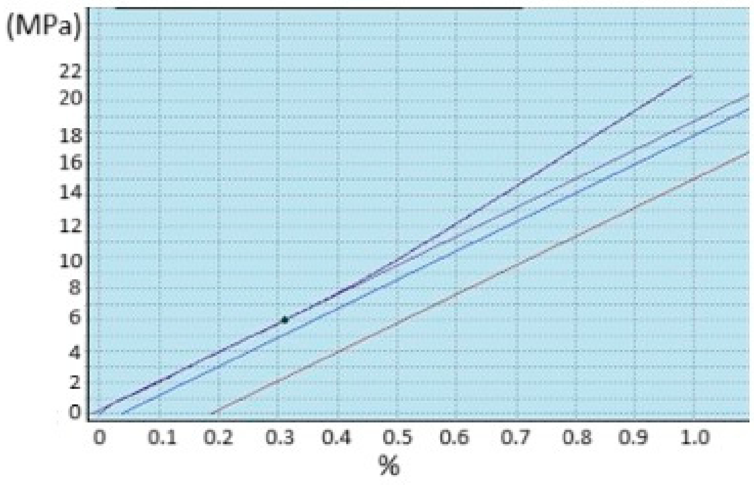


**Figure S3.** Static tensile test graph for Epidian 57/ Z1 adhesive.

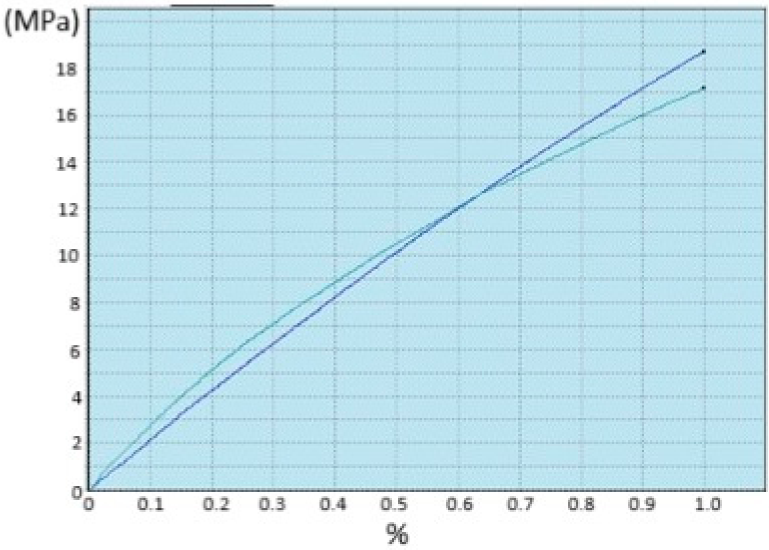


**Figure S4.** Static tensile test graph for 3M DP420 adhesive.

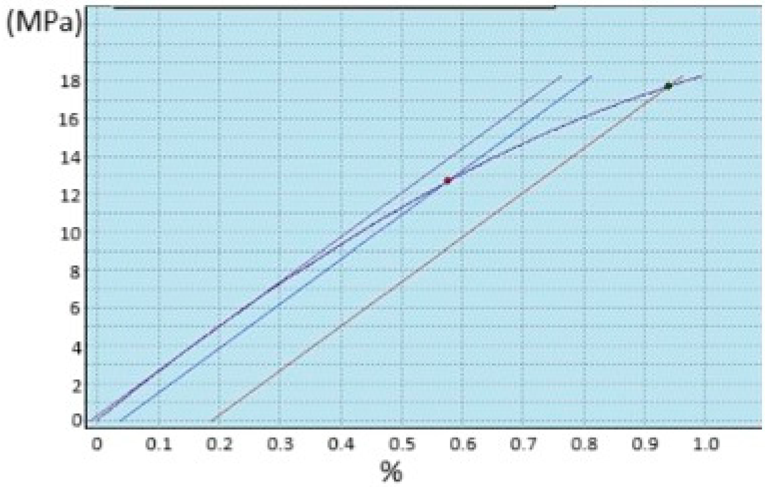


**Figure S5.** Static tensile test graph for EA9464 adhesive.

Adhesive's samples were stretched on the HT-2402 Hung Ta universal testing machine with a measurement range of up to 100 kN. An extensometer Epsilon 3542 with a measuring base of 25 mm was used for the tests. The graphs were drawn by the machine's software.
